# Combined statistical-biophysical modeling links ion channel genes to physiology of cortical neuron types

**DOI:** 10.1016/j.patter.2025.101323

**Published:** 2025-08-05

**Authors:** Yves Bernaerts, Michael Deistler, Pedro J. Gonçalves, Jonas Beck, Marcel Stimberg, Federico Scala, Andreas S. Tolias, Jakob H. Macke, Dmitry Kobak, Philipp Berens

**Affiliations:** 1 Hertie Institute for AI in Brain Health, University of Tübingen, 72076 Tübingen, Germany; 2 Tübingen AI Center, 72076 Tübingen, Germany; 3 Champalimaud Centre for the Unknown, Champalimaud Foundation, 1400-038 Lisbon, Portugal; 4 Department of Computer Science, University of Tübingen, 72076 Tübingen, Germany; 5 VIB-Neuroelectronics Research Flanders (NERF), Leuven, Belgium; 6 Department of Computer Science, KU Leuven, 3001 Leuven, Belgium; 7 Department of Electrical Engineering, KU Leuven, 3001 Leuven, Belgium; 8 Sorbonne Université, CNRS, Institut des Systèmes Intelligents et de Robotique, 75005 Paris, France; 9 Baylor College of Medicine, Houston, TX 77030, USA; 10 Department of Ophthalmology, Byers Eye Institute, Stanford University, Stanford, CA 94303, USA; 11 Department of Empirical Inference, Max Planck Institute for Intelligent Systems, 72076 Tübingen, Germany

**Keywords:** simulation-based inference, Hodgkin-Huxley model, biophysics, cell types, model misspecification, single-cell transcriptomics, electrophysiology, mouse motor cortex

## Abstract

Neurons have classically been characterized by their anatomy, electrophysiology, and molecular markers. More recently, single-cell transcriptomics has enabled an increasingly fine genetically defined taxonomy of cortical cell types, but the link between the gene expression of individual cell types and their physiological and anatomical properties remains poorly understood. Here, we develop a hybrid modeling approach to bridge this gap: our approach combines statistical and mechanistic models to predict cells’ electrophysiological activity from gene expression patterns. To this end, we fit Hodgkin-Huxley-based models for a wide variety of cortical cell types by using simulation-based inference while overcoming the mismatch between model and data. Using multimodal Patch-seq data, we link the estimated model parameters to gene expression using an interpretable linear sparse regression model. Our approach identifies the expression of specific ion channel genes as predictive of biophysical model parameters including ion channel densities, implicating their mechanistic role in determining neural firing properties.

## Highlights

• Gene expression predicts ion channel conductances in cortical neuron models
• Biophysical models bridge transcriptomic data to electrophysiology
• Robust simulation-based inference overcomes model-data mismatch

## The bigger picture

The brain contains an extraordinary diversity of neuronal cell types, each with distinct genetic signatures and functional properties. While recent advances in single-cell transcriptomics have allowed us to classify neurons with unprecedented resolution, a central question remains: how do gene expression patterns give rise to the rich diversity of neuronal behaviors? This work addresses this question by introducing a hybrid modeling approach that combines biophysical simulations with modern machine learning techniques. The authors develop interpretable conductance-based models of neuronal activity, fitting them to electrophysiological recordings from hundreds of neurons. They then use these models as an intermediate layer to statistically link gene expression to electrophysiological properties through sparse regression techniques. The approach is noteworthy because it bridges data-driven statistical models and mechanistic simulations, providing a new way to interpret complex multimodal data such as Patch-seq. It also introduces a modified inference method that remains robust in the face of imperfect models, a common challenge in biological systems. By revealing mechanistic links between genes and function, this study moves us closer to a causal understanding of neuronal identity. The framework is general and can be applied to other tissues or systems where linking gene expression to physiology is essential, making it a valuable contribution for systems neuroscience, computational biology, and data-driven biophysics.

## Teaser

This study introduces a hybrid statistical-biophysical framework that links gene expression to neuronal electrophysiology using mechanistic Hodgkin-Huxley models and simulation-based inference. By analyzing Patch-seq data from mouse cortical neurons, the authors uncover predictive relationships between ion channel gene expression and biophysical parameters, offering new mechanistic insights into how genetic identity shapes neuronal function.

## Introduction

Neural cell types form the basic building blocks of the nervous system.^1^ In the neocortex, they form intricate circuits giving rise to perception, cognition, and action.^2^^,^^3^^,^^4^ Scientists have classically characterized these cell types by their anatomy or electrophysiology, and, more recently, using molecular markers.^3^^,^^5^^,^^6^^,^^7^ In the past decade, single-cell transcriptomics has enabled an increasingly fine genetically defined taxonomy of cortical cell types,^8^^,^^9^^,^^10^^,^^11^ but the link between the gene expression profiles of individual cell types and their physiological and anatomical properties remains poorly understood.

To tackle this question, Patch-seq has been developed to combine electrophysiological recordings, single-cell RNA sequencing, and morphological reconstruction in individual neurons.^12^^,^^13^^,^^14^^,^^15^ This approach has made it possible to directly study the relationship between the gene expression profile of a neural cell type and its physiological and anatomical characteristics. These studies have found that distinct families of neurons (such as *Pvalb* or *Sst* interneurons or intratelencephalic pyramidal neurons) show distinct physiological and anatomical properties.^16^^,^^17^ Within these families, cellular properties often vary continuously,^17^ possibly caused by smooth changes in gene expression.

This wealth of data has led to the development of sophisticated techniques for multimodal data integration and analysis,^18^^,^^19^^,^^20^^,^^21^ but uncovering the mechanistic relationships between, e.g., transcriptomic and physiological properties of neurons has been challenging. For example, sparse reduced-rank regression (sRRR) can reveal patterns of ion channel gene expression statistically predictive of particular expert-defined electrophysiological features^17^ but precludes a potential causal interpretation. Establishing mechanistic links experimentally is challenging as well, as it involves genetic or pharmacological interventions.

**Figure 1:**
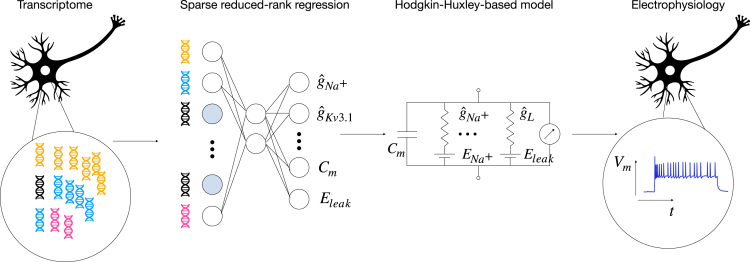
Sketch of the statistical-mechanistic hybrid model Neuronal gene expression levels (left) and electrophysiological recordings (right) are obtained experimentally with Patch-seq. The neuronal responses to electrical stimulation are fit with a conductance-based biophysical model (middle right). The estimated model parameters are then predicted with a sparse reduced-rank regression model from the gene expression data (middle left).

Here, we argue that biophysical models of the physiological activity of neurons can help to close this gap as their parameters are explicitly interpretable (Figure 1, right). We constructed conductance-based models with single compartments^22^^,^^23^^,^^24^ for the electrophysiological activity of 955 neurons from the adult mouse motor cortex (MOp) spanning various neural types and classes.^17^ In contrast to the expert-defined features previously used to relate gene expression and physiological response patterns, the parameters of these models correspond to mechanistically interpretable quantities such as ion channel densities. We then applied sRRR to predict the fitted conductance-based model parameters from the gene expression patterns in the same set of cells, completing the statistical-biophysical bridge of gene expression to electrophysiological activity with mechanistically interpretable latent quantities (Figure 1).

To find parameters of the mechanistic model that explain observed electrophysiology, we used neural posterior estimation (NPE).^25^^,^^26^^,^^27^ This approach can recover the parameters of a mechanistic model based on summary statistics derived from model simulations, providing a posterior distribution over the model parameters. The posterior distribution allows to quantify the uncertainty in our model parameter estimates, in contrast to previous work using genetic algorithms^28^^,^^29^ and much more efficiently than Markov chain Monte Carlo (MCMC)-based approaches.^30^ As has been observed in other contexts,^31^^,^^32^^,^^33^^,^^34^ we found that NPE failed for our biophysical model and dataset due to a small but systematic mismatch between the data and the model, which could not be easily remedied by standard modifications to the model. We developed an algorithm that introduces noise to the summary statistics of model simulations used to train the density network, which allowed it to perform reliable inference despite the model misspecification.

Using this new algorithm, we obtained posteriors over the conductance-based model parameters for all 955 neurons in our diverse dataset. We found that parameter samples from the posterior provided good model fits to the physiological firing patterns of most neurons but observed higher parameter uncertainty in some families such as *Vip* interneurons. Furthermore, we showed that the relationship between gene expression patterns and the inferred model parameters could be learned using statistical techniques such as sRRR, allowing to predict the electrophysiology of a cell from its gene expression across cortical neuron types and classes. Our approach recovered specific ion channel genes as predictive of model parameters corresponding to matching ion channel densities, directly implicating them in a mechanistic role for determining specific neuronal firing patterns that differ between cell types.

## Results

### Hodgkin-Huxley-based models reproduce Patch-seq electrophysiological recordings

To better understand how the genetic identity of a cortical neuron determines its physiological activity, we studied a previously published dataset of neurons from the mouse motor cortex, which had been characterized with respect to their gene expression profile and electrophysiological properties using Patch-seq^17^ (see methods). We focused on a subset of 955 cells that displayed action potentials to injection of a step current of 300pA and passed transcriptomic quality control. These neurons had non-zero expression of 7.2 thousand genes on average (ranging between 1.2 and 18.1 thousand genes). The dataset included 278 pyramidal neurons and 677 interneurons, consisting of 289 *Pvalb*, 240 *Sst*, 54 *Vip*, and 11 *Sncg* neurons, using the cell families and finer cell type labels assigned by the original authors^17^ based on mapping the gene expression patterns of the neurons to a larger reference atlas.^11^ To test the generalization of our modeling framework, we additionally applied it to a dataset of n=4,107 interneurons from the mouse visual cortex.^16^

We hypothesized that we could further clarify the relationship between gene expression patterns and the electrophysiological response properties of the neurons, if we knew the mechanistically interpretable biophysical parameters underlying those. Therefore, we implemented a single-compartment conductance-based model based on a previously established “minimal” Hodgkin-Huxley (HH)-based model that captures the electrophysiology in a wide range of neuronal families.^23^ We then increased the flexibility of our model with the addition of a small number of ion channels important for modeling pyramidal cells.^24^ The parameters of the resulting model included passive parameters such as the capacitance and input resistance as well as active conductances of different ion channels or currents, which determine the physiological responses (see methods). Our final model included different sodium ($Na^{+}$), potassium ($K^{+}$), calcium ($Ca^{2+}$), and leak currents and had 13 free parameters overall, which needed to be inferred from experimental recordings. We summarized experimental recordings by 23 expert-defined electrophysiological features, including latency to the first spike; action potential count, amplitude, and width; and the membrane potential mean and variance (for a full list of all 23 features, see methods), and computed the same features for each HH model simulation.

**Figure 2:**
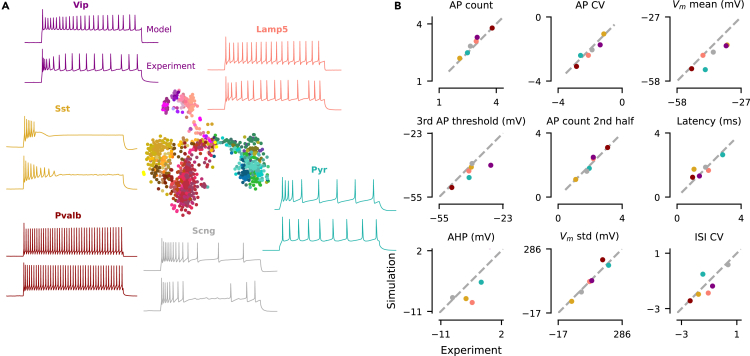
Measurements from all major neuronal families can be simulated by the HH-based model (A) Middle: t-SNE embedding of n=955 MOp cells based on their transcriptome. Surround: one example neuron for each of the six transcriptomic families. For each neuron, we show the experimental observation (bottom) and the biophysical model simulation from the prior with the smallest Euclidean distance in standardized electrophysiological feature space (top). Colors correspond to the six families of cortical neurons, *Pvalb* in red, *SSt* in yellow, *Vip* in purple, *Lamp5* in rosé, *Scng* in gray, and *pyramidal* cells in green. Color variations within these families correspond to the cell types of Yao et al.^11^ (B) Comparison of nine electrophysiological feature values between experimental observations and best prior simulations shown in (A). Average (with SD) Euclidean distance from observations to best simulations is shown in Table 1, in the prior row.

Our HH-based model was able to generate simulations that were close to experimental observations from all major families of neurons, both qualitatively and quantitatively. We defined a uniform prior distribution over the 13 free parameters within biologically plausible ranges, sampled 15 million parameter combinations from it, and ran the biophysical simulation for each of them with the Brian2 toolbox.^35^ Out of the 15 million simulations, about 7 million had well-defined values for each of the 23 electrophysiological features. For a given Patch-seq neuron, we picked the parameter combination yielding the simulation lying closest (in terms of Euclidean distance) to the actual neuron in standardized electrophysiological feature space (each feature was *Z* scored with the mean and standard deviation of 7 million simulations). This simulation was typically qualitatively similar (Figures 2 and S1) and matched experimental electrophysiological features well (Table 1, last row). However, this strategy required a very large library of precomputed prior simulations and yields only a point estimate, i.e., the best fitting model parameter vector, and no uncertainty information.

**Table 1:** Performance of various NPE training approaches

Training	MAP		Posterior	
	Fails (%)	Eucl. distance	Fails (%)	Eucl. distance
Standard NPE	12.77	6.24±3.24	17.69	6.63±3.24
Best Euclidean	14.14	5.11±3.20	20.86	5.70±3.36
0.001 SD noise to ephys	7.33	5.36±3.10	12.58	5.43±3.17
0.01 SD noise to ephys	5.86	4.81±2.96	8.54	5.39±3.11
0.05 SD noise to ephys	4.61	4.71±2.98	9.01	5.34±3.06
0.1 SD noise to ephys **(NPE-N)**	2.51	4.35±2.86	**6.22**	**5.11**±**3.03**
1 SD noise to ephys	**1.36**	**3.81**±**2.11**	7.27	5.19±2.56
0.05 SD noise to ephys and model params	3.66	5.42±2.91	10.28	6.31±2.86
Data augmentation	3.35	4.47±2.98	6.98	4.91±3.12
Prior	0.00	2.63±0.81	52.29	11.79±3.31
Standard NPE, V1	7.13	6.04±15.66	11.58	6.40±14.25
0.1 SD noise to ephys **(NPE-N)**, V1	**1.31**	**3.78**±**2.16**	**3.55**	**4.63**±**2.57**
Prior, V1	0.00	3.69±1.48	52.29	11.79±3.31

For the descriptions of training approaches, see methods. The columns show the percentage of simulation fails (at least one undefined summary statistic) and the Euclidean distance to the experimental values (mean ± SD over n=955 MOp neurons) using MAP parameters and using 10 randomly drawn samples from the NPE posterior. In the row for the prior distribution, we take the prior simulation closest to the experimental observation and sample 10 parameter combinations from the prior. Bold values show best rows in each column (excluding the prior row). Final three rows show NPE, NPE-N, and prior performance on a second mouse visual (V1) cortex dataset (see methods).

### Neural posterior estimation with noise

Therefore, we used neural posterior estimation (NPE),^25^^,^^26^^,^^36^ an algorithm for simulation-based inference,^37^ which learns an approximate posterior distribution $q\left(\theta |x_{o}\right)$ over the parameter vector θ, given a vector of features $x_{o}$ computed from the experimental recording (see methods). The posterior distribution is parameterized as a sufficiently flexible neural density estimator, namely, a masked autoregressive normalizing flow.^38^ Given a biologically informed uniform prior distribution (see methods, Table 2), the posterior distribution $q\left(\theta |x_{o}\right)$ quantifies the probability that a parameter set θ generates summary features that *exactly* match the summary features of the experimental recordings $x_{o}$. In contrast to previous methods,^28^^,^^29^ this approach allowed us to quantify the uncertainty in the parameters after seeing the data. We trained the neural density estimator on our synthetic dataset comprising 7 million simulations from our HH-based model to infer the 13 free parameters, given the 23 electrophysiological features. After training, the neural network could be evaluated on features of *any* experimental recording and could return the corresponding posterior distribution without further simulations or training, providing a model of this relationship.

**Table 2:** Description of the 13 HH-based model parameters

Model parameter	Prior range	Description
*C*	$\left[0.1,15\right]\frac{\mu F}{cm^{2}}$	the membrane capacitance *C* measures how much charge can be stored per voltage difference $V_{m}$ across the membrane
$R_{input}$	[20,1000]MΩ	the input resistance $R_{input}$, equals the membrane voltage $V_{m}$ deflection from resting state divided by injected current. The inverse is called the leak conductance $g_{leak}$
*τ*	[0.1,70]ms	here, *τ* describes the time for the membrane potential to *increase* by a fraction of (1−1/e), or 63%, from its resting membrane state during the application of the positive 300pA current pulse
${\bar{g}}_{Nat}$	$\left[0,250\right]\frac{\text{mS}}{cm^{2}}$	maximal conductance of the fast inactivating $Na^{+}$ current^24^^,^^39^
${\bar{g}}_{Na}$	$\left[0,100\right]\frac{\text{mS}}{cm^{2}}$	maximal conductance of the $Na^{+}$ current^23^^,^^40^
${\bar{g}}_{Kd}$	$\left[0,30\right]\frac{\text{mS}}{cm^{2}}$	maximal conductance of the delayed rectifier $K^{+}$ current^23^^,^^40^
${\bar{g}}_{M}$	$\left[0,3\right]\frac{\text{mS}}{cm^{2}}$	maximal conductance of the slow non-inactivating muscarinic $K^{+}$ current^23^^,^^41^
${\bar{g}}_{Kv3.1}$	$\left[0,250\right]\frac{\text{mS}}{cm^{2}}$	maximal conductance of the fast non-inactivating $K^{+}$ current^24^^,^^42^
${\bar{g}}_{L}$	$\left[0,3\right]\frac{\text{mS}}{cm^{2}}$	maximal conductance of the high-threshold $Ca^{2+}$ current^23^^,^^43^
$E_{leak}$	[−130,−50]mV	reversal potential of the leak current
$\tau_{\max}$	[50,4000]ms	time constant describing how rapid the muscarinic current channel opens (see $g_{M}$)
$V_{T}$	[−90,−35]mV	parameter that can adjust the AP threshold
$r_{SS}$	[0.1,3]	rate to steady state (SS). Parameter introduced to change how rapid gates reach open and closed steady states in $Na^{+}$ ion channel with maximal conductance $\bar{g}$_Na_ and $K^{+}$ ion channel with maximal conductance ${\bar{g}}_{Kd}$

**Figure 3:**
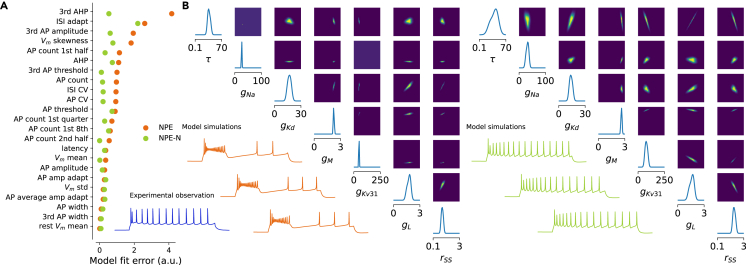
NPE vs. NPE with noise (A) The MAP parameter set simulation derived with NPE-N is closer to the experimental reference (in blue below, *L4/5 IT_1* pyramidal cell) than derived with NPE. Residual distance of the MAP parameter set simulation to the experimental observation (model fit error) shown for each electrophysiological feature (0 corresponds to a perfect fit). (B) One- and two-dimensional marginals together with 3 simulations generated from parameter combinations with highest probability under the posterior (out of 10,000 samples); NPE (left) vs. NPE-N (right) setting. Seven out of 13 model parameters have been selected for illustration.

**Figure 4:**
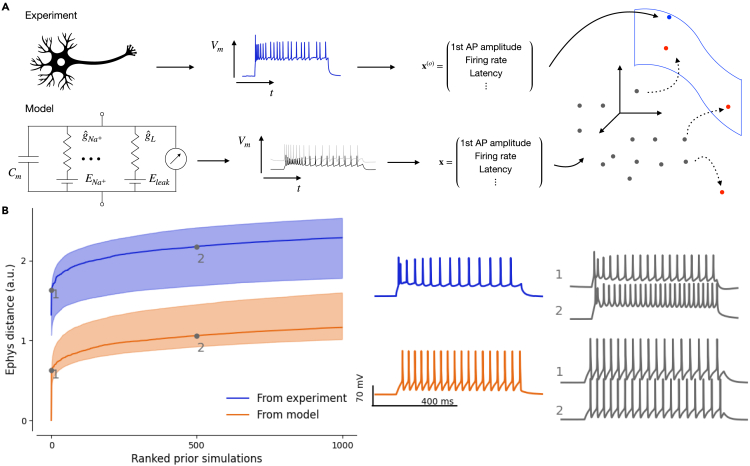
NPE of conductance-based model parameters in the presence of model misspecification (A) Sketch illustrating model misspecification: in electrophysiological feature space, not enough simulations cover the space of experimental observations. NPE-N introduces isotropic noise to the summary statistics of simulations (dotted arrows). (B) Simulations are further away from experimental observations (blue) than from other simulations (orange). Qualitatively, simulations increasingly further away from an experimental observation look more dissimilar than from another simulation. Numbers 1 and 2 refer to the 2nd and 501st closest simulations, respectively.

However, this procedure did not work well for the neurons in our dataset. In many cases, samples from the posterior or the maximum a posteriori (MAP) parameters did not produce simulations that came close to the experimental data (Figures 3 and S2). Moreover, 18% of posterior-sampled parameters produced simulations with at least one undefined summary feature, for example, undefined latency due to complete lack of action potentials (Table 1). We investigated the reason for this failure and found that the poor performance of the inference framework was due to a systematic mismatch between the electrophysiological recordings and simulations from the model (Figure 4A), a phenomenon recently observed also in other settings.^31^^,^^32^^,^^33^^,^^34^ For a given simulation, only few other simulations were very close to it in the feature space, but even simulations lying further away still produced qualitatively very similar outcomes (Figure 4B, orange). In contrast, for a given experimental trace, the distance to the closest simulation was much larger, even when qualitatively the fit looked reasonable (Figure 4B, blue). We found similar results in the additional visual cortex dataset (Figure S3).

We concluded that the experimental observations occupied a region of the electrophysiological feature space that was systematically shifted from the region occupied by the model simulations (Figure 4A), despite our best efforts to create an HH-based model that has sufficient flexibility to capture the response diversity of the cortical neurons in our dataset (Figure 2). For instance, we introduced the $r_{SS}$ parameter to the model (see methods), which can scale the speed with which ion channels reach open steady states, in order to alleviate model-data mismatches observed in the action potential width, which were especially large in pyramidal cells and difficult for the model to capture without it. Although this approach substantially reduced overall model mismatch, it did not remove it entirely (Figure S4). As a consequence, many simulations produced with randomly drawn posterior parameters had either one or more undefined electrophysiological feature or found themselves at a large distance to their experimental reference (Figure 3A; Table 1).

We found that modifying the NPE algorithm improved the performance of the inference procedure. To improve the density estimator’s generalization to experimental observations at test time, we smoothed the feature space by adding a small amount of independent Gaussian noise to the electrophysiological features of selected simulations close to the experimental observations and used those to train the neural density estimator (Figure 4A and methods). This procedure yielded posterior simulations that came much closer to their experimental reference both qualitatively and quantitatively (Figure 3). The Euclidean distance in electrophysiological feature space between the experimental recording and the simulation with the MAP biophysical parameters $∥x_{\text{MAP}}-x∥$ was 4.35±2.86 when using noise vs. 6.24±3.24 when using standard NPE (mean ± SD across n=955 cells; Table 1). We experimented with several strategies of adding noise of different magnitude and chose a compromise between simulations from the posterior being close to the measured data and a low fraction of simulations that result in undefined features (see methods). We called the resulting procedure neural posterior estimation with noise (NPE-N) and used it to obtain posterior distributions over parameters for all 955 neurons in our dataset. NPE-N outperformed NPE both qualitatively and quantitatively across various cell types as showcased for six additional cells representative of different cell types in Figures S5–S10. We also applied NPE and NPE-N on the additional visual cortex dataset and found comparable results (Table 1; Figure S2).

To gain further insights into the inference procedure, we asked which of the 23 features were most important to constrain the posterior. To this end, we used an algorithm that efficiently compares a posterior constrained by the full set of features to one constrained by a growing subset of features^44^ and studied a subset of 50 neurons (see methods). We ran this algorithm five times for each of these neurons and counted how often a feature was selected as one of the five most important features (Figure S11A). We then compiled the results of this selection procedure across all 50 neurons and found that, on average, the mean resting membrane potential was by far the single most important feature, followed by the mean potential during current stimulus, action potential amplitude, the action potential threshold, and the variance of the membrane potential (Figure S11B).

### Transcriptomic, electrophysiological, and HH-based model parameter variability

**Figure 5:**
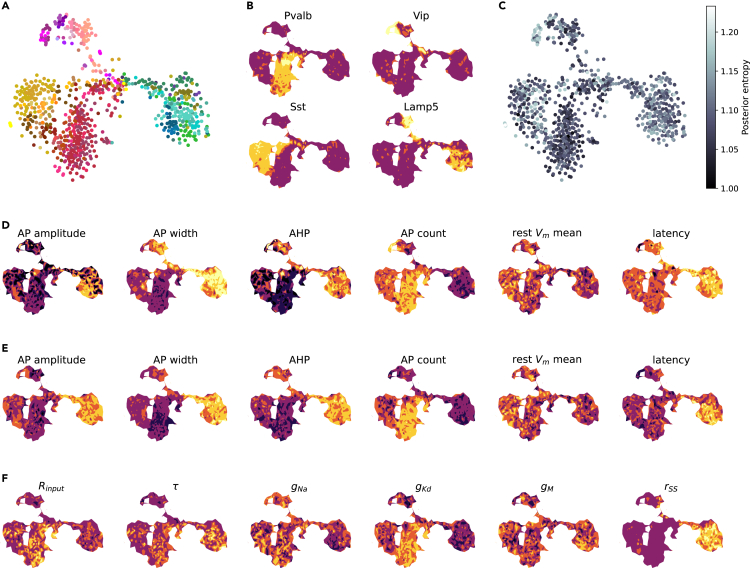
Two-dimensional embedding reveals difference in HH-based parameters between neural families (A) T-SNE embedding of n=955 MOp neurons based on transcriptomic data. Colors correspond to the six families of cortical neurons, *Pvalb* in red, *SSt* in yellow, *Vip* in purple, *Lamp5* in rosé, *Scng* in gray, and *pyramidal* cells in green. Color variations within these families correspond to the cell types of Yao et al.^11^ Cells in the middle of the embedding had lower quality transcriptomic data and therefore grouped together. (B) Marker gene expression levels overlaid and interpolated on embedding confirm known families (dark purple, low expression; yellow, high expression). (C) Uncertainty of MAP parameters for each cell overlaid on the embedding. The uncertainty was calculated as the posterior entropy $-\sum_{k=1}^{1000}\;\log\;q_{\phi }\left(\theta_{k}|x_{o}\right)$, where we sampled $\theta_{k}∼q_{\phi }\left(\theta |x_{o}\right)$ and then normalized by the cell with least entropy. (D) Selection of summary statistics derived from simulations corresponding to MAP estimates, overlaid on the embedding. (E) Selection of summary statistics describing observed electrophysiology, overlaid on the embedding. (F) Selection of MAP parameters, overlaid on the embedding.

We next returned to our original question and studied how the transcriptomic identity of the neurons in our dataset was related to their electrophysiological properties and the MAP parameters of the best-fitting HH-based model (Figures 5 and S12). To this end, we used a two-dimensional t-distributed stochastic neighbour embedding (t-SNE) visualization of the gene expression data of all 955 MOp neurons (Figure 5A). We found that the embedding separated the major neural families, including interneurons and pyramidal neurons, well (Figure 5A). We confirmed the identity of these families by overlaying the expression strength of various marker genes such as *Pvalb*, *Sst*, *Vip*, and *Lamp5* (Figure 5B). The NPE-N posteriors for neurons from some families were less constrained than those of others, indicated by higher posterior entropy (Figure 5C). Specifically, this affected *Vip* neurons, which were relatively sparsely sampled in the dataset. In contrast, *Pvalb* neurons showed the lowest uncertainty indicating that their posteriors were best constrained using the available features. One reason for this may be that *Pvalb* neurons fired more stereotypically, whereas *Vip* neurons showed greater variability in their firing patterns,^17^^,^^45^ which may require greater flexibility in the model to reproduce.

We overlaid the individual electrophysiological features on this two-dimensional embedding, both for the simulated MAP traces and experimentally measured data (Figures 5D and 5E). We found that, as expected, these features varied strongly between neural families and that the features extracted from simulated traces matched well to the features from measured traces. For example, pyramidal neurons showed higher action potential amplitude and width as well as lower firing rates, in line with the observations. For some features, such as the latency of the response, the match was less perfect, as the simulated traces of interneuron families had overall higher latency than those experimentally measured.

Next, we studied how the parameters of the HH-based model varied across this transcriptomically defined embedding (Figure 5F). This visualization allowed us to reason about the relationship between biophysical parameters and the resulting electrophysiological properties in some of the genetically defined families. For example, the conductivity of the delayed rectifier potassium current ${\bar{g}}_{Kd}$^40^ was estimated to be high for *Pvalb* interneurons (Figure 5F), suggesting that these currents were important for quickly repolarizing the membrane potential $V_{m}\left(t\right)$ during action potential (AP) generation in order to obtain the small AP widths and high AP count of fast-spiking *Pvalb* cells (Figures 5D–5F). Likewise, the membrane time constant *τ* and the scaling parameter $r_{SS}$ were important to fit the large action potential widths and high latency observed for pyramidal neurons (Figures 5D–5F). These findings were corroborated in the additional visual cortex dataset (Figure S13).

We further investigated how well neural families and types could be distinguished based on the MAP parameter estimates for the HH model. To this end, we trained a logistic regression on 80% of all cells and tested them on the remaining 20% of cells. We found that the cell family was classified correctly for 75.4% of the cells, while fine cell types could only be correctly assigned in 24.6% of neurons.

### Closing the gap: From genes to electrophysiology

**Figure 6:**
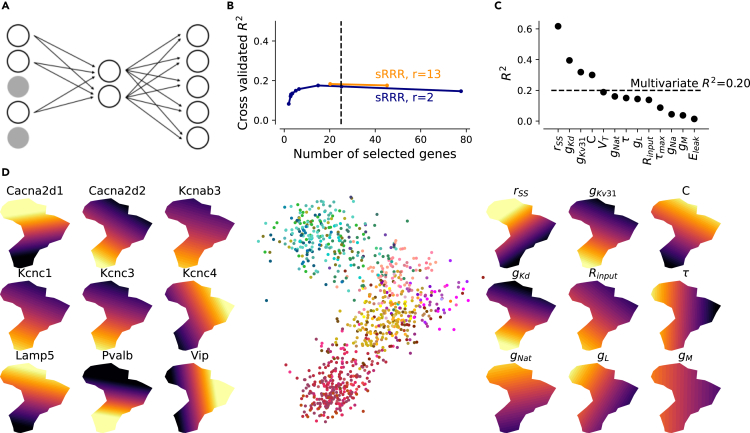
Prediction of MAP parameter estimates from gene expression with sRRR (A) sRRR schematic. A linear combination of selected genes is used to predict fitted HH-based model parameters. Full gray circles denote genes that were not selected by the linear model. (B) Cross-validation performance for rank-2 and full-rank sRRR models with elastic net penalty. The dashed vertical line shows the performance with 25 genes. (C) Rank-2 sRRR model predictive performance for each model parameter, using the entire dataset. Colors correspond to the six families of cortical neurons, *Pvalb* in red, *SSt* in yellow, *Vip* in purple, *Lamp5* in rosé, *Scng* in gray, and *pyramidal* cells are shown in green. Color variations within these families correspond to the cell types of Yao et al.^11^ (D) Middle: rank-2 sRRR model latent space visualization. All 955 MOp neurons are shown. Left: selected ion channel and marker gene overlays. Right: predicted model parameter overlays.

Given these results, we were now in a position to develop a quantitative model relating the transcriptomic identity of a neuron and its biophysical parameters. To this end, we trained a linear sparse reduced-rank regression (sRRR) model^18^ and a nonlinear sparse bottleneck neural network (sBNN)^19^ to predict the biophysical parameters (d=13) from the gene expression data (Figure 6A). To ease the interpretability, we focused on ion channel and known marker genes only (d=427) and trained linear and nonlinear models with a two-dimensional latent space. We found that model parameters could be predicted with reasonable accuracy (sRRR: $R^{2}=0.17\pm 0.03$, mean ± SD across cross-validation folds, for a model selecting approximately 25 genes) and that the nonlinear model performed just as well as the linear one (sBNN: $R^{2}=0.17\pm 0.03$), so we analyzed only the linear model further (Figure 6B). Over the entire dataset, this model predicted some parameters such as the conductance of the fast non-inactivating and delayed rectifying potassium channel (${\bar{g}}_{KV3.1}$ and ${\bar{g}}_{Kd}$) or the membrane capacitance *C* particularly well (Figure 6C). Other model parameters were less well predicted, such as the leak potential $E_{leak}$ or the muscarinic potassium channel conductance ${\bar{g}}_{M}$. Interestingly, the $r_{SS}$ parameter, which we introduced as the first step toward alleviating model mismatch issues, was predicted the best.

We visualized the latent space of the sRRR model to better understand the relationship between ion channel and marker genes and the HH-based model parameters (Figure 6D). This embedding is conceptually similar to the t-SNE visualization of the entire gene space with overlaid model parameters and electrophysiological properties (Figure 5), except that here we focus on genes that predict HH-based model parameters. The two-dimensional latent space of the sRRR model showed two principal directions of variation, where one separated pyramidal cells from interneurons and the other distinguished different interneuron families. In addition, we found that the sRRR model identified mechanistically plausible relationships: for example, the potassium channel conductances ${\bar{g}}_{Kv3.1}$ and ${\bar{g}}_{Kd}$ were both high in *Pvalb* neurons placed in the lower left corner, predicted by the expression of various potassium channel genes such as *Kcnc1*, which constitutes a subunit of the Kv3.1 voltage-gated potassium channel, and *Kcnab3*, respectively. Likewise, the calcium channel conductance ${\bar{g}}_{L}$ was predicted by high expression of *Cacna2d1*, which directly encodes the alpha-2 and delta subunits in the L-type calcium channel. Our sRRR model selected *Cacna2d2* as well, which is a paralog gene with opposite expression to *Cacna2d1* (Figure 6D, left). In addition, classical marker genes such as *Vip* acted as surrogate cell family markers and contributed to the prediction. Analysis of the visual cortex dataset revealed similar marker genes for families including *Kcnc2*, *Cacna2d1*, and *Vip* as predictive of fitted model parameter values (Figure S14).

**Figure 7:**
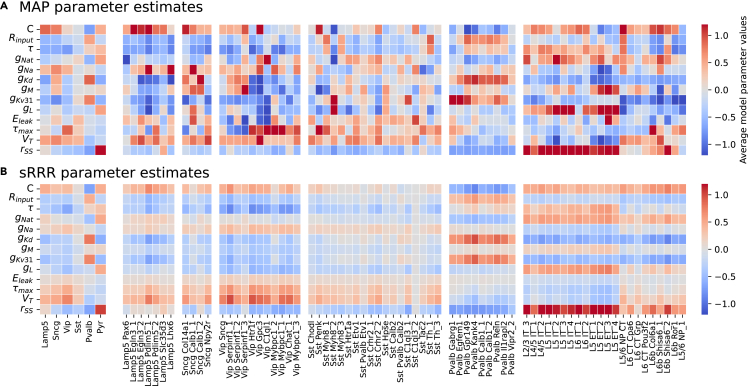
MAP parameter estimates and sRRR predictions for each family and cell type (A) MAP parameter estimates averaged over cells belonging to a family and belonging to a transcriptomic cell type (left and right, respectively). We *Z* scored all values by subtracting and scaling with the mean and standard deviation of n=955 MAP estimates, respectively. (B) Analogous to (A) but with rank-2 sRRR-predicted model parameter values.

**Figure 8:**
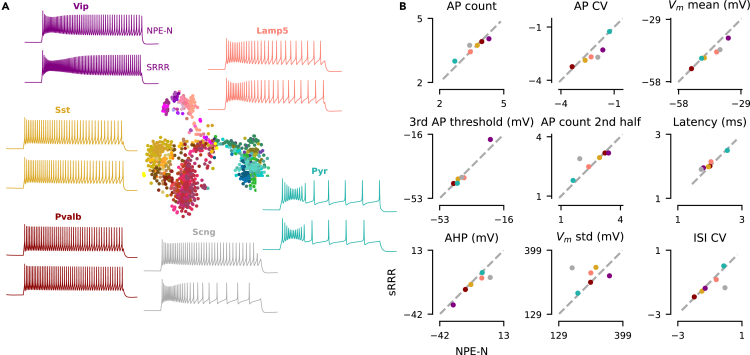
Family representation of MAP estimates together with sRRR predictions (A) Analogous to Figure 2A, except that the simulation on top is derived from the family-average MAP estimate calculated as in Figure 7A, left. Simulation on the bottom is derived from the family-average sRRR prediction calculated as in Figure 7B, left. Colors correspond to the six families of cortical neurons, *Pvalb* in red, *SSt* in yellow, *Vip* in purple, *Lamp5* in rosé, *Scng* in gray, and *pyramidal* cells in green. Color variations within these families correspond to the cell types of Yao et al.^11^ (B) Comparison of nine electrophysiological feature values derived with the MAP estimate versus sRRR-based estimate.

This approach can be used to predict HH-based models for neurons for which we only measured gene expression but not electrophysiology, especially on the family level. The model predictions captured essential variation in the model parameters on the family level, although less so on the cell-type level (Figure 7). To quantify this, we measured the Euclidean distance (normalized by variance) between the matrix with average NPE-fitted MAP parameter values and sRRR-predicted parameter values (Figures 7 and S15). On the family level, this distance was substantially smaller than on the cell-type level—18.02 (family level, left) vs. 50.15, 41.94, 103.21, 118.29, 28.70, and 126.02 (for *Lamp5*, *Sncg*, *Vip*, *Sst*, and *Pvalb* interneurons and pyramidal cells, respectively, right)—indicating that the variation in gene expression levels can be used to predict electrophysiology accurately on a family level but less so on the cell-type level. In agreement, a logistic regression classifier trained on the sRRR predictions performed as well on the family and cell-type levels (accuracy 75.0% vs. 26.0%) as the logistic regression classifier directly trained on the HH parameters, indicating that, overall, sRRR predictions retained the information about families and cell types present in the fitted HH parameters. Within pyramidal neurons, sRRR also captured some variability between major groups: a logistic regression classifier trained to distinguish intratelencephalic/extratelencephalic (IT/ET) pyramidal neurons from the remaining types with ≈80% accuracy from MAP parameters and sRRR predictions. Also qualitatively, an HH-based model simulated with HH parameters predicted by sRRR matched well those simulated with the average MAP parameters. Except for *Scng* interneurons for which we had only few (n=11) cells available, sRRR-based predictions matched the electrophysiological feature values of MAP-based model predictions and generated simulations almost indistinguishable by eye (Figures 8 and S13).

## Discussion

In this study, we directly linked the gene expression profile of a set of cortical neurons to HH-based model parameters fitted to their electrophysiological signatures. We believe this is a major step toward a more mechanistic understanding of how a neuron’s gene expression profile determines its electrophysiology from previous work, where we and others have simply correlated gene expression and electrophysiology, e.g., predicting electrophysiological properties from transcriptomic data.^17^^,^^18^^,^^19^^,^^20^^,^^21^

The mechanistic HH-based model we used here spells out our understanding of how electrophysiological properties arise from ion channel densities and other passive properties of a neuron, leaving the link between these quantities and the expression of certain genes to be explained by a statistical model. In our approach, we used a linear reduced-rank regression model with groupwise sparsity constraint on the genes, selecting genes in or out of the model based on the available data. Given the present data, we found that the linear model with a two-dimensional intermediate layer performed as well as a comparable nonlinear model. Partially, this may be due to the noise in gene expression and the comparably small dataset, but it is also possible that our explicit mechanistic model for the generation of electrophysiological activity explained away some of the nonlinear relationship between gene expression and electrophysiological features. Much larger datasets will likely also help the linear model to resolve better observed differences in model parameters between fine cell types, which are currently captured only to a certain extent (Figure 7).

Previous work has also attempted to infer biophysical parameters in HH-based models and link the inferred values to neural cell classes and their gene expression.^23^^,^^24^^,^^28^^,^^29^ Unlike our work, most of these studies did not directly link parameters in HH-based models to the expression of a large set of genes but rather studied parameter differences between genetically defined cell classes.^23^^,^^24^^,^^28^ One recent study examined the relationship between HH-based model parameters and individual genes^29^ but did not provide a predictive model for this link as their work is based on two *separate* datasets, a large one with single-cell transcriptomic data and a smaller one with morphological and electrophysiology data. To link gene expression to HH-based model parameters, they use the fact that both datasets were acquired in the same *cre* transgenic lines, a link that is known to be tenuous.^46^ In contrast, we provide a direct statistical model for the relationship between gene expression and fitted model parameters. Also, none of these previous studies used uncertainty-aware parameter inference techniques. Incorporating uncertainty allowed us to highlight cells, cell types, or families for which the inference procedure returned results that were not as well constrained by the data. Furthermore, this uncertainty-aware method showed the well-known parameter degeneracy in HH-based models,^25^^,^^47^^,^^48^ as marginals of the posterior distribution covered extended regions of parameter space. Alternative approaches capture uncertainty in the posterior based on MCMC sampling,^30^ but this required running MCMC for each cell with hundreds of iterations and dozens or hundreds of sampling chains. Instead, NPE-N requires training a neural network once on a large simulated training dataset, which can then be used to predict the posterior distribution for any observation without further simulations or retraining.

Many trends in model parameters across different cell classes qualitatively matched previous observations. For instance, we found that different values are needed for the potassium conductance ${\bar{g}}_{Kv3.1}$ to model *Vip*, *Sst*, and *Pvalb* neurons and that the expression of *Kcnc1* varies accordingly (Figure 6D). In a similar vein, Nandi et al. report different values for ${\bar{g}}_{Kv3.1}$ and show that *Kcnc1* is differentially expressed between these cell families.^29^ We found that our predictive sRRR model successfully captured model parameter differences between major neural families but struggled with differences between finer cell types. In addition, we found it significantly more difficult to predict a neuron’s cell type than its family identity from NPE-N-derived model parameter values. There could be multiple reasons for this finding. The dataset used could be too small, but applying our pipeline to a three times larger mouse visual cortex dataset^16^ did not change this finding, such that only much larger datasets may help. Another potential reason is that electrophysiological properties at the cell-type level vary continuously and subtly within the major families,^17^ suggesting that it could be difficult to capture them with a statistical model based on transcriptomic data of individual neurons with data at this scale. Finally, the inherent noise of transcriptomic data on the single-cell level^49^ could contribute to this limitation as well.

We found that a systematic mismatch between our simulations and the experimental data caused out-of-the-box simulator-based inference methods to fail. We first attempted to fix the domain gap by introducing the $r_{SS}$ parameter to improve the used HH model for the wider AP widths observed in pyramidal cells, which helped to alleviate some of the model mismatch that we originally observed. This parameter adapted the rate with which $Na^{+}$ and $K^{+}$ ion channel gates reach their respective steady state in the model (Table 2), effectively changing the dynamics of in- and outflow of these ions and therefore the AP width. Potentially, additional channels^50^ or a more complicated multi-compartment morphology with inhomogeneous spatial distribution of channels could further narrow the domain gap and better explain the physiology of some of the neurons.

We overcame the challenge of the remaining domain gap by adding noise to the summary statistics derived from the simulations, effectively smoothing the feature space. In parallel work to this paper, this phenomenon has recently received more widespread attention^31^^,^^32^^,^^33^^,^^34^: for instance, robust NPE^31^ takes the opposite approach to our strategy and denoises the measured data toward the model using Monte Carlo sampling. On the other hand, robust synthetic likelihood approaches^51^ that estimate likelihoods rather than posteriors work similarly to our approach. Which strategy works best for which models and circumstances remains to be evaluated, but these strategies will allow to apply simulation-based inference techniques in cases where models provide relatively coarse but useful approximations of the true phenomena. Alternatively, one could make the model more realistic. In our case, some of the model mismatch is likely also caused by the use of single-compartment models in contrast to other studies that used HH-based models with two or more compartments^24^^,^^28^^,^^29^; however, such complex models are currently difficult to use with simulation-based inference.

Mechanistic models that make biophysical processes explicit are ultimately desirable all the way from gene expression to electrophysiology, as such models form the highest level of causal understanding.^52^ To further close this causality gap would require an explicit mechanistic model for the translation of mRNA into proteins such as ion channels—a relationship that is all but simple.^53^^,^^54^^,^^55^ Mechanistic models for this process have been suggested in the literature on a variety of scales,^56^^,^^57^ but it is an open question how such models could be integrated in the inference procedure, given the temporal and spatial processes involved in mRNA translation.^56^^,^^57^^,^^58^ While directly measuring translation dynamics in live cells has become possible using live-cell imaging approaches,^59^^,^^60^ it remains an extremely challenging task, especially in multicellular systems and given the diversity of cortical neurons. Therefore, the combination of machine learning-based models with explicit mechanistic models may provide a viable path forward to improve our understanding of neuronal diversity even if we do not have full causal empirical knowledge of the entire chain of events, aiding the inference of important intermediate quantities.

## Methods

### Dataset

We reanalyzed a published dataset consisting of n=1,328 adult mouse motor cortex (MOp) neurons,^17^ which had been characterized transcriptomically and electrophysiologically using Patch-seq. We downloaded the read count data from GitHub, https://github.com/berenslab/mini-atlas, and the electrophysiological traces from DANDI: 000008, https://dandiarchive.org/dandiset/000008. The authors used Smart-seq2 to obtain single-cell transcriptomes for these neurons. Out of n=1,328 cells, n=1,213 cells passed transcriptomic quality control and were assigned a transcriptomic cell type using the 1,000 genes that were most variable across this subset of cells. For electrophysiological characterization, the authors injected negative to positive constant currents for 600 ms time windows starting at −200 pA with steps of 20 pA to positive currents beyond 400 pA or until the cell died. Electrophysiological experiments were performed at a temperature of 25°C. For further experimental details, see Scala et al.^17^ Finally, out of n=1,328 cells, we analyzed n=955 cells that had well-defined summary statistics in their membrane voltage response to current injection of 300 pA.

### HH-based model

We used a single-compartment HH-based model^23^ that was designed to reproduce electrophysiological behavior of a wide variety of neurons across species with a minimal set of ion channels. To account for the variability across excitatory and inhibitory cortical neurons, we added additional ion channels^24^ and introduced $r_{SS}$, a parameter influencing how rapid gates reach open and closed steady states in some sodium and and potassium currents. Without these modifications, we could not fit wider AP widths observed in pyramidal cells.

The HH-based model solves the following ordinary differential equation (ODE) $V_{m}\left(t\right)=f\left(V_{m}\left(t\right),\theta \right)$ for $V_{m}\left(t\right)$, the membrane voltage as a function of time:

$$\frac{dV_{m}\left(t\right)}{dt}=\frac{1}{C}\left(I_{Na}+I_{Nat}+I_{Kd}+I_{M}+I_{Kv3.1}+I_{L}+I_{leak}-I_{inj}-I_{noise}\right)$$

$$I_{Na}={\bar{g}}_{Na}m^{3}h\left(E_{Na^{+}}-V_{m}\left(t\right)\right)$$

$$I_{Nat}={\bar{g}}_{Nat}{\hat{m}}^{3}\hat{h}\left(E_{Na^{+}}-V_{m}\left(t\right)\right)$$

$$I_{Kd}={\bar{g}}_{Kd}n^{4}\left(E_{K^{+}}-V_{m}\left(t\right)\right)$$

$$I_{M}={\bar{g}}_{M}p\left(E_{K^{+}}-V_{m}\left(t\right)\right)$$

$$I_{Kv3.1}={\bar{g}}_{Kv3.1}v\left(E_{K^{+}}-V_{m}\left(t\right)\right)$$

$$I_{L}={\bar{g}}_{L}q^{2}r\left(E_{Ca^{2+}}-V_{m}\left(t\right)\right)$$

$$I_{leak}={\bar{g}}_{leak}\left(E_{leak}-V_{m}\left(t\right)\right)\text{.}$$

Here, ${\bar{g}}_{x}$ and $E_{x}$ denote the maximum channel conductance and reversal potential of membrane ion channel *x*, respectively. *C* is the membrane capacitance, and $I_{inj}=300\;\text{pA}$ denotes the magnitude of experimental current injected between current stimulation onset at 100ms and stimulation offset 700ms. In order to model small membrane voltage fluctuations observed experimentally, we further introduced Gaussian current noise $I_{noise}∼N\left(10\;\text{pA},1\;\text{pA}\right)$ at every time point.

Furthermore, ion channel activation and inactivation gates follow dynamics $\frac{dx}{dt}=\alpha_{x}\left(V_{m}\left(t\right)\right)\left(1-x\right)+\beta_{x}\left(V_{m}\left(t\right)\right)x$, where $x\in \left\{m,h,\hat{m},\hat{h},n,p,v,q,r\right\}$. Opening $\alpha_{x}$ and closing $\beta_{x}$ rate constants depend on the membrane voltage $V_{m}\left(t\right)$, as previously described.^23^^,^^24^ To account for the 25°C temperature at which Patch-seq experiments were performed, we used a temperature coefficient $Q_{10}=2.3$ to scale the kinetics with which gates in ion channels open and close. The parameter $r_{SS}$ further scales the rates with which sodium and potassium currents with maximal conductances ${\bar{g}}_{Na}$ and ${\bar{g}}_{Kd}$ reach steady states.

In total, 13 parameters in the model can be tuned in order to fit observed electrophysiology of n=955 MOp cells (see Table 2 for details). We implemented the model with the Brian2 toolbox^35^ in Python, which can efficiently transpile and simulate models in C*++*.

To understand the significance of adding the $r_{SS}$ in reducing model misspecification on a “model implementation” level rather than training the density estimator, we compared with the same HH-based model but with scaling parameter $r_{SS}=1$ (Figure S4).

### Electrophysiological features

We automatically extract 23 electrophysiological summary features from the experimental or simulated voltage traces V(t) using a Python library from the Allen Software Development Kit (https://github.com/AllenInstitute/AllenSDK) with modifications to account for our experimental paradigm (https://github.com/berenslab/hh_sbi; Table 3).

**Table 3:** Description of 23 extracted electrophysiological features

Electrophysiological feature	Description
AP threshold	membrane voltage at the time where the first derivative of the voltage w.r.t. time reaches a threshold, which elicits the 1st AP
AP amplitude	height of the 1st AP, measured from threshold to maximum voltage
AP width	width at half height of the 1st AP
AHP	afterhyperpolarization. Depth of the membrane voltage drop after the 1st AP, measured from AP threshold
3rd AP threshold	analogous to AP threshold but for the 3rd AP
3rd AP amplitude	analogous to AP amplitude but for the 3rd AP
3rd AP width	analogous to 3rd AP width but for the 3rd AP
3rd AHP	analogous to AHP but for the 3rd elicited AP
AP count^a^	number of elicited APs in the current injection window 100−700ms
AP counts 1st 8th^a^	number of elicited APs in 100−175ms
AP count 1st quarter^a^	number of APs in 100−250ms
AP count 1st half^a^	number of APs in 100−400ms
AP count 2nd half^a^	number of APs in 400−700ms
AP amp adapt^a^	AP amplitude adaptation. 1st elicited AP amplitude divided by the amplitude of the 2nd elicited AP
AP average amp adapt^a^	AP average amplitude adaptation. Average ratio of all two consecutive AP heights as calculated by AP amp adapt during current injection window
AP CV^a^	coefficient of variation (SD divided by the mean) of all AP amplitudes of APs elicited during the current injection window
ISI adapt^a^	interspike interval (ISI) adaptation. ISI: time elapsed between two APs. ISI adapt: ratio of the 2nd ISI (between 2nd and 3rd elicited AP) to the 1st ISI (between 1st and 2nd elicited AP)
ISI CV^a^	coefficient of variation (SD divided by the mean) of all ISIs
Latency^a^	time it takes to elicit the 1st AP, measured from current stimulation onset to AP threshold
Rest $V_{m}$ mean	mean of the membrane voltage $V_{m}$ before current stimulation onset 0−100ms. Also called resting membrane potential
$V_{m}$ mean	mean of the membrane voltage $V_{m}$ during current stimulation window 100−700ms
$V_{m}$ SD	SD of the membrane voltage $V_{m}$ during current stimulation window 100−700ms
$V_{m}$ skewness	skewness of the membrane voltage $V_{m}$ during current stimulation window 100−700ms

a To make their distribution more Gaussian, these features are additionally log transformed, except for the AP average amp adapt, for which we used the sigmoid transform.

### Standard NPE

Standard NPE applied to HH-based models starts with sampling model parameter sets from a prior distribution θ∼p(θ), followed by creating synthetic datasets through the simulator or HH-based model, and eventually trains a deep neural density estimator $q_{\phi }\left(\theta |x\right)$, more specifically in our case, a (masked autoregressive) normalizing flow,^38^^,^^61^ to learn the probabilistic association between summary statistics x derived from simulations and its parameter sets θ. Experimental observations $x_{o}$ can then be fed to the density estimator in order to derive all parameter sets consistent with the data and the prior, i.e., the posterior distribution $q_{\phi }\left(\theta |x_{o}\right)$.^25^

We used the sbi toolbox https://sbi-dev.github.io/sbi to run NPE with different training schedules, including NPE-N, which we explain in the next section.

### Dealing with model mismatch in NPE

Posterior distributions derived with standard NPE can suffer from model mismatches, that is, when simulations generally fail to adequately cover experimental observations in summary statistic or electrophysiological space. They can become confidently wrong, placing high posterior weight on parameter sets that do not reproduce experimental recordings and low posterior weight to parameter sets that do (Figure 3B, left). In machine learning jargon, the trained density estimator fails to extrapolate to experimental observations (test data) that are outside of the distribution of the training data.

We experimented with various modifications of NPE in order to make the posterior more robust to the mismatch between model and experimental observations. First, we tried to include only simulations that position themselves close to experimental observations in summary statistic space (Table 1, best Euclidean). Closeness was measured by calculating the Euclidean distance between simulation and observation after standardizing all summary statistics. Second, we introduced different levels of isotropic Gaussian noise to the summary statistics of those simulations (Table 1, x SD noise to ephys). Third, besides adding noise to summary statistics, we introduced isotropic Gaussian noise to the model parameters with which the close simulations were generated (Table 1, 0.05 SD noise to ephys and model parameters). Finally, we experimented with a mixture of non-manipulated close simulations and close simulations with noise added to their summary statistics (Table 1, data augmentation).

Given their performance measures (Table 1), we decided to use NPE with added isotropic Gaussian noise only to the summary statistics of simulations close to experimental observations in summary statistic space. We call the method NPE-N. The noise is of moderate amplitude such that a tradeoff is established between closeness of simulations of MAP estimates to the experimental observations with closeness of simulations from random posterior samples (Table 1, 3rd and 4th column).

In contrast to NPE, NPE-N produced posteriors that give high posterior weight to model parameter sets that both qualitatively and quantitatively produce simulations close to experimental observations.

### Feature selection through likelihood marginalization

To analyze which features were informative for constraining the inference procedure, we used feature selection through likelihood marginalization^44^ (FSLM). To ensure comparable posterior estimates between FSLM and NPE, we trained FSLM with 3 million spiking simulations randomly generated from the prior to which we also introduced isotropic Gaussian noise in their summary statistics. We then drew 1,000 samples from the posteriors of each observation, $x_{o}$, for 5 different initializations of FSLM and only selected the 50 experimental observations with smallest average $KL\left(p_{NPE-N}\left(\theta |x_{o}\right)|p_{FSLM}\left(\theta |x_{o}\right)\right)$^62^ for feature selection. The final ranking was derived from 1,000 samples per posterior and is averaged across 10 initializations.

### Visualization

We used the openTSNE^63^ implementation with default parameters to embed the transcriptomic space with t-SNE to a final two-dimensional representation.

### sRRR and sparse bottleneck neural networks

To link gene expression data to HH-based model parameters, we used sRRR.^18^ This linear statistical tool reduces the high-dimensional gene space data to a two-dimensional latent (rank = 2), which is maximally predictive of the model parameters. An elastic net penalty was used to select the most relevant genes. As a nonlinear extension to sRRR, we also tested the use of nonlinear sparse bottleneck neural networks (sBNNs) that utilize a neural network with bottleneck to predict electrophysiological measurements from transcriptomic space.^19^ Analogously to sRRR, a group lasso penalty was used on the weights of the first layer to select most meaningful genes.

### Mouse visual cortex dataset

We extracted electrophysiological feature values (Table 3) of n=4,107 mouse visual cortex cells with raw recordings available at DANDI: 000020, https://dandiarchive.org/dandiset/000020/. In the study of Gouwens et al.,^16^ electrophysiological recordings were conducted with various current stimulation paradigms, including ramps, short (3ms) current pulses, as well as long (1s) current steps. To make a meaningful comparison to this study, we derived electrophysiological feature values from membrane voltage responses corresponding to the 1s long current steps. We could not find responses to 300pA current steps consistently for all cells as in our study and so allowed the current step to vary across cells. Consequently, we introduced one more current step parameter in the HH-based model (see methods) in order to fit their interneuron recordings from mouse visual cortex. Importantly, their recordings were conducted at 34°C (instead of 25°C), and cells were bathed in internal solutions containing 10mM phosphocreatine disodium salt hydrate (instead of 5mM). The latter implied setting the Nernst potential for sodium to $E_{Na^{+}}=53.5\;\text{mV}$ instead of $E_{Na^{+}}=69.0\;\text{mV}$ (ours). We found other non-inferred parameter values such as the Nernst potential for potassium and calcium to be similar to ours.

As we introduced one more biophysical parameter, we simulated HH-based models from 20 million (instead of 15 million) different parameter combinations sampled from the prior (see methods). Similarly to our synthetic dataset of simulations, we then experimented with various training paradigms for NPE (see methods).

Finally, we found n=3,559 cells with matching transcriptome in their dataset (see NeMO Archive, https://data.nemoarchive.org/other/AIBS/AIBS_patchseq/transcriptome/scell/SMARTseq/processed/analysis/20200611/) and used those to predict MAP parameter estimates derived for each cell with NPE-N from their gene expression levels with sRRR.

## Resource availability

### Lead contact

Requests for further information and resources should be directed to and will be fulfilled by the lead contact, Philipp Berens (philipp.berens@uni-tuebingen.de).

### Materials availability

No new materials have been generated in this study.

### Data and code availability

• Raw electrophysiological recordings are publicly available at DANDI: 000008, https://dandiarchive.org/dandiset/000008/. Further preprocessed data are available either directly in the code repository for this study at GitHub, https://github.com/berenslab/hh_sbi, or on Zenodo, https://doi.org/10.5281/zenodo.7716391. Read counts can be downloaded from GitHub, https://github.com/berenslab/mini-atlas.
• Code to train density neural networks, analyze their performance, and produce figures in this manuscript can be found on GitHub, https://github.com/berenslab/hh_sbi, and has also been deposited on Zenodo, https://zenodo.org/records/15463046^64^. This code builds upon the simulation-based inference package *sbi*^27^ (https://sbi-dev.github.io/sbi), the simulator package *Brian2* (https://brian2.readthedocs.io/en/stable/), automatic ephys feature extraction pipeline (https://github.com/berenslab/EphysExtraction), parallel processing package *Pathos*^65^ (https://mmckerns.github.io/project/pathos/wiki.html), and *openTSNE*^63^ (https://github.com/pavlin-policar/openTSNE).
• Refer to the lead contact for further requests.

## Acknowledgments

We thank Ziwei Huang for discussion. We thank the Deutsche Forschungsgemeinschaft (Heisenberg Professorship BE 5601/8-1 and Excellence Cluster 2064 “Machine Learning—New Perspectives for Science,” ref. 390727645). The work was also funded by the 10.13039/501100000780European Union (ERC, “NextMechMod,” ref. 101039115 and “DeepCoMechTome,” ref. 101089288). Views and opinions expressed are, however, those of the authors only and do not necessarily reflect those of the European Union or the European Research Council Executive Agency. Neither the 10.13039/501100000780European Union nor the granting authority can be held responsible for them. Additional support comes from the 10.13039/100000025National Institute of Mental Health and 10.13039/100000065National Institute of Neurological Disorders and Stroke under award no. U19MH114830. The content is solely the responsibility of the authors and does not necessarily represent the official views of the National Institutes of Health. This work was also supported by the 10.13039/100000025National Institute of Mental Health grant UM1 MH130981 and by the 10.13039/100000002NIH under award no. R01 MH109556.

## Author contributions

Conceptualization, P.B. and Y.B.; methodology, Y.B., M.D., P.J.G., J.B., J.H.M., D.K., and P.B.; software, Y.B. and M.S.; formal analysis, Y.B.; data curation, Y.B. and F.S.; writing – original draft, Y.B. and P.B.; writing – review & editing, all authors; visualization, Y.B.; supervision, A.S.T., J.H.M., D.K., and P.B.; project administration, P.B.; funding acquisition, A.S.T., J.H.M., and P.B.

## Declaration of interests

The authors declare no competing interests.

## Supplemental information

Document S1. Figures S1–S15

Document S2. Article plus supplemental information
