# Supplementary material for: Combined statistical-biophysical modeling links ion channel genes to physiology of cortical neuron types
Source: Patterns (N Y). 2025 Aug 5;6(10):101323. doi: 10.1016/j.patter.2025.101323 (PMC12546760; doi:10.1016/j.patter.2025.101323)
Supplement: Document S1. Figures S1–S15 [file mmc1.pdf]

Patterns, Volume *6*

Supplemental information

Combined statistical-biophysical modeling links ion channel genes to physiology of cortical neuron types

Yves Bernaerts, Michael Deistler, Pedro J. Gonçalves, Jonas Beck, Marcel Stimberg, Federico Scala, Andreas S. Tolias, Jakob H. Macke, Dmitry Kobak, and Philipp Berens

Supplementary Figures 1–15

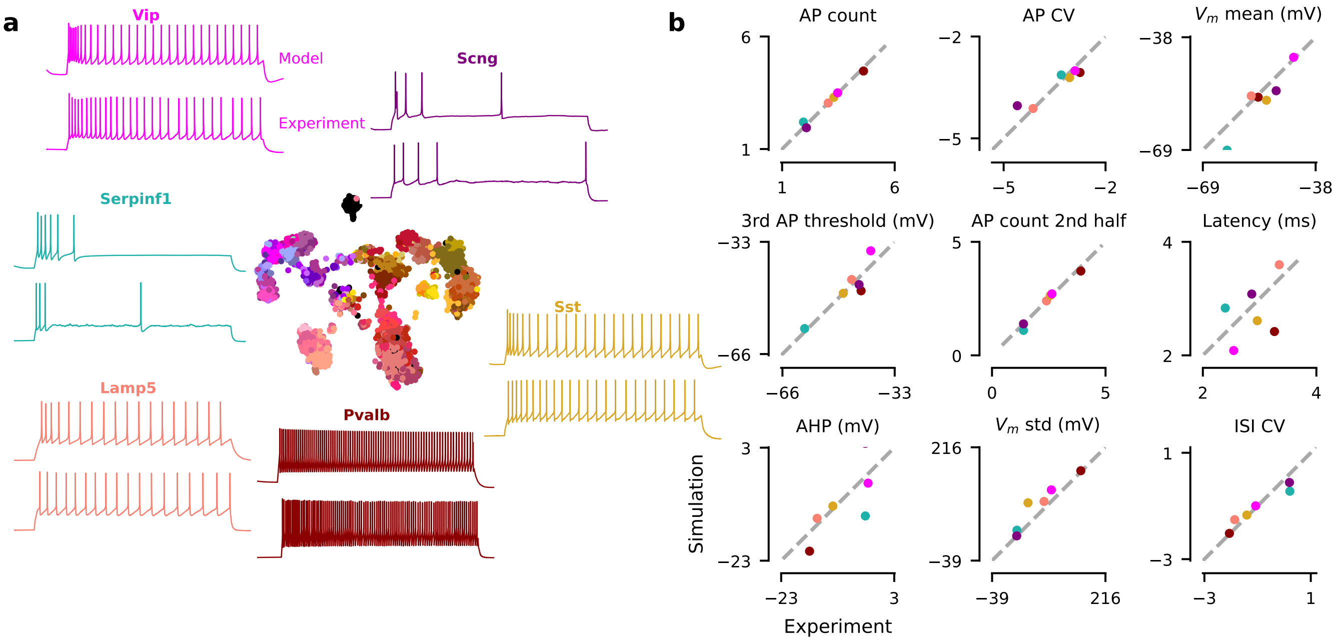

Figure S1 **Example experimental observations and their closest simulations from the prior.** Analogous to Fig. 2, but for mouse visual cortex [16]. T-SNE embedding from $n = 3559$ interneurons with transcriptome (see Methods).

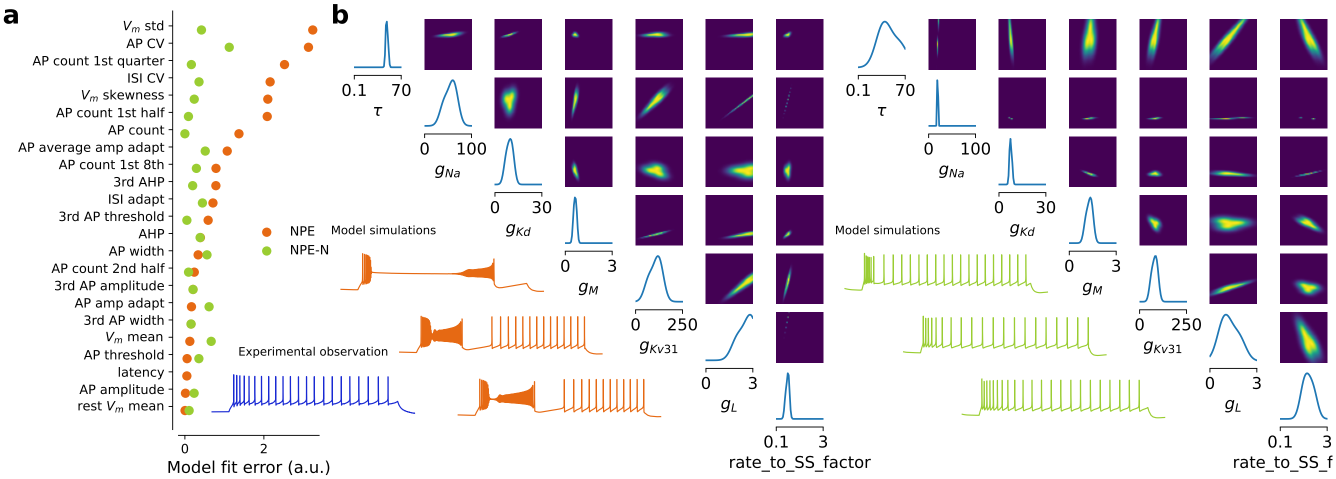

Figure S2 **Neural Posterior Estimation vs Neural Posterior Estimation with Noise.** Analogous to Fig. 3, but for mouse visual cortex [16].

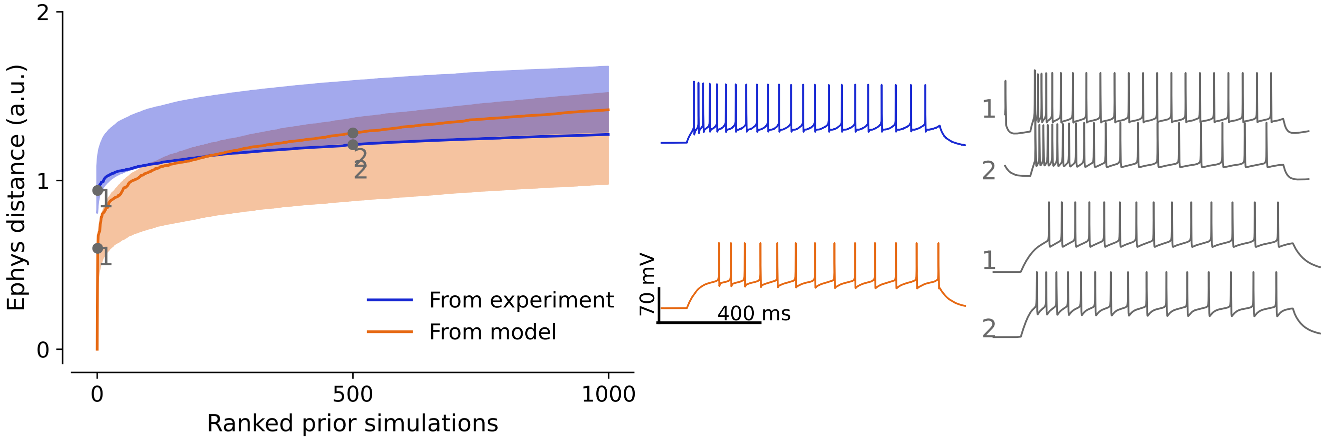

Figure S3 **Neural posterior estimation of conductance-based model parameters in the presence of model misspecification.** Analogous to Fig. 4, but for mouse visual cortex[16].

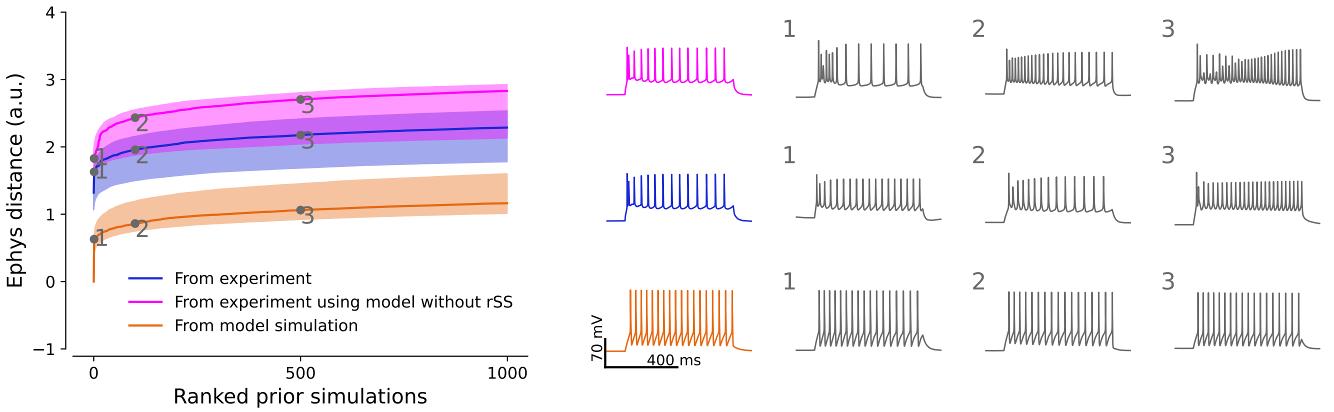

Figure S4 Model misspecification with and without scaling r_{SS} parameter. Analogous to Fig. 4, but including model simulations without r_{SS} parameter.

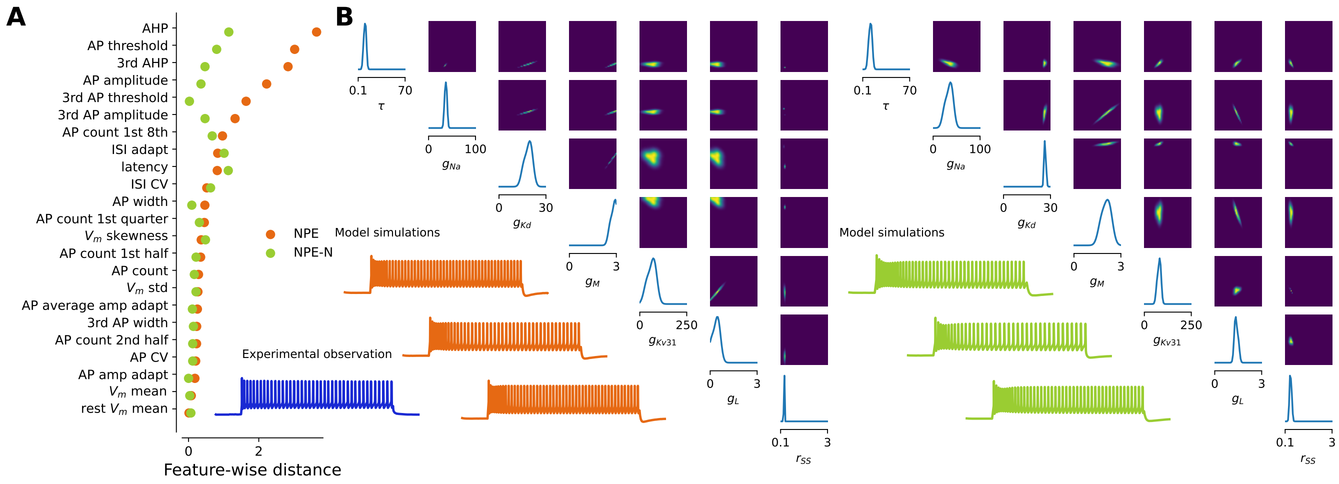

Figure S5 NPE vs NPE-N, illustration 1: fast-spiking *Pvalb Calb1_1* interneuron. Analogous to Fig. 3.

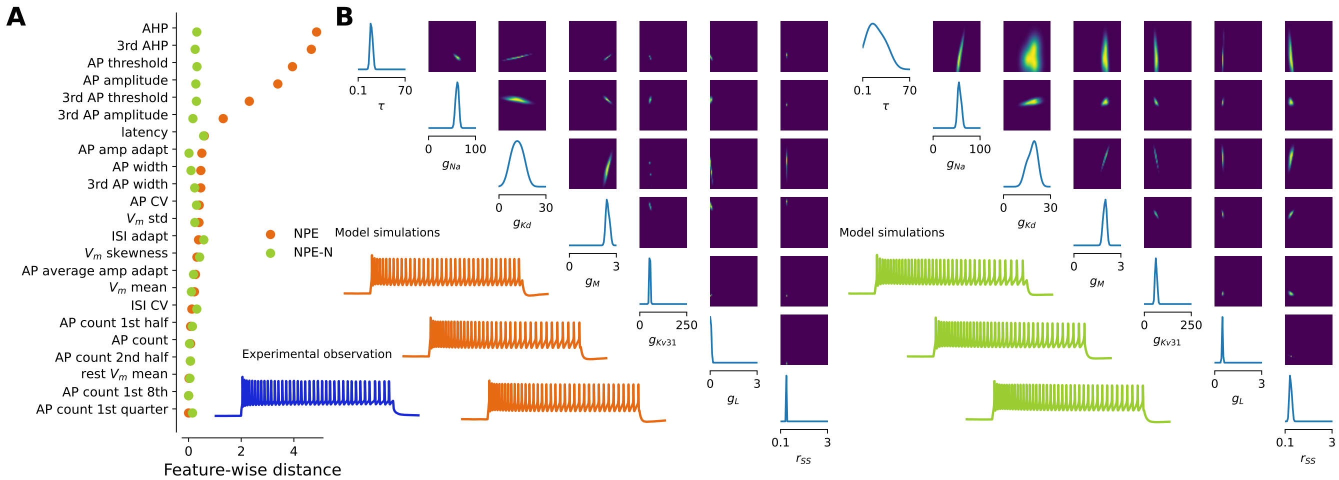

Figure S6 **NPE vs NPE-N, illustration 2:** ***Sst Crhr2_1*** **interneuron.** Analogous to Fig. 3.

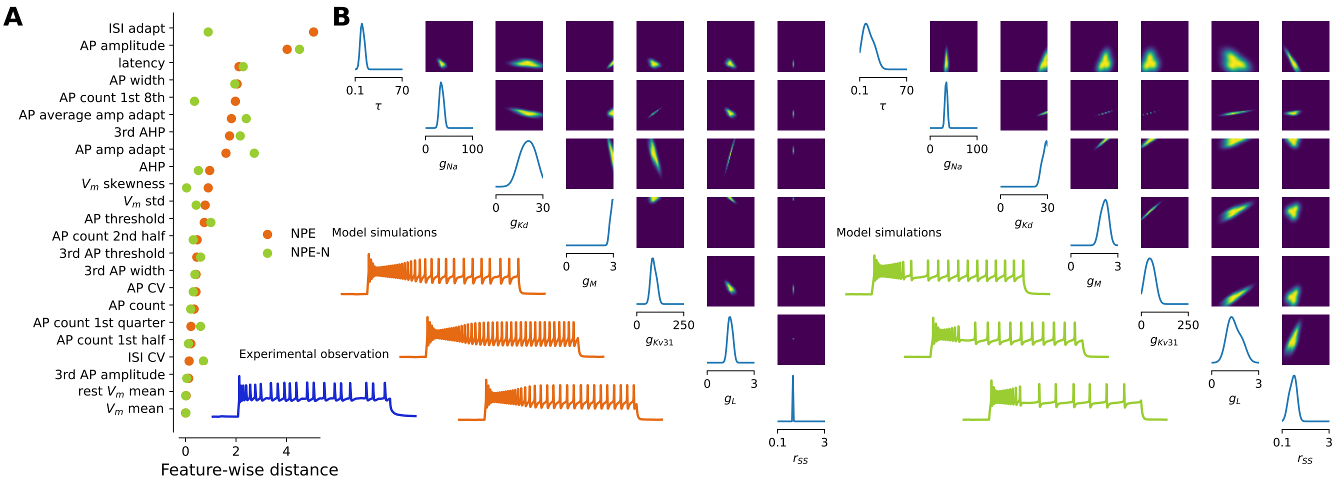

Figure S7 **NPE vs NPE-N, illustration 3:** ***Vip Serpinf1_1*** **interneuron.** Analogous to Fig. 3.

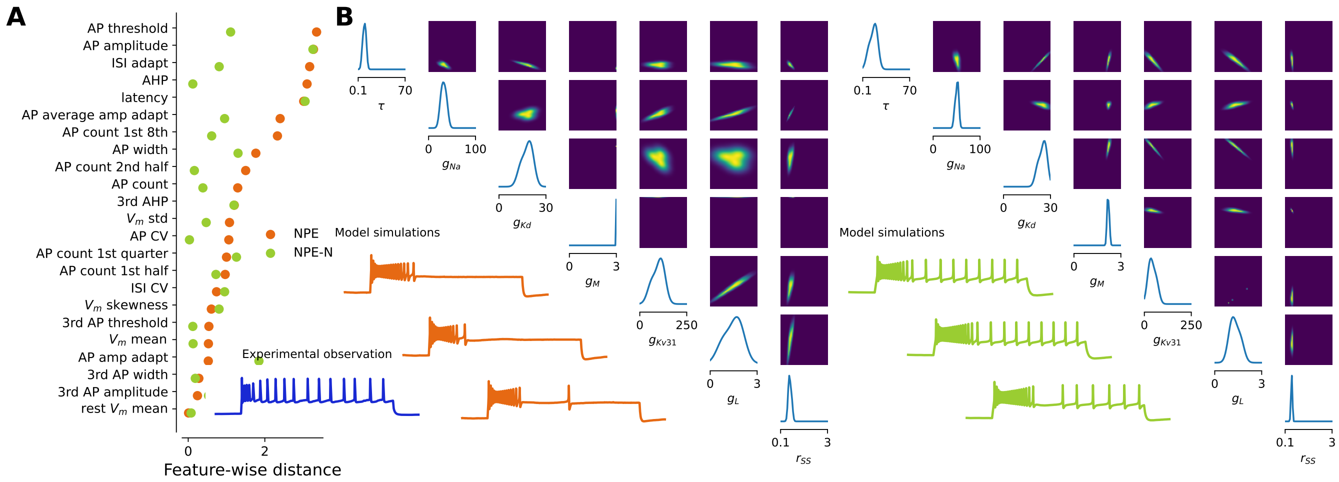

Figure S8 **NPE vs NPE-N, illustration 4:** ***Lamp5 Egln3_1*** **interneuron.** Analogous to Fig. 3.

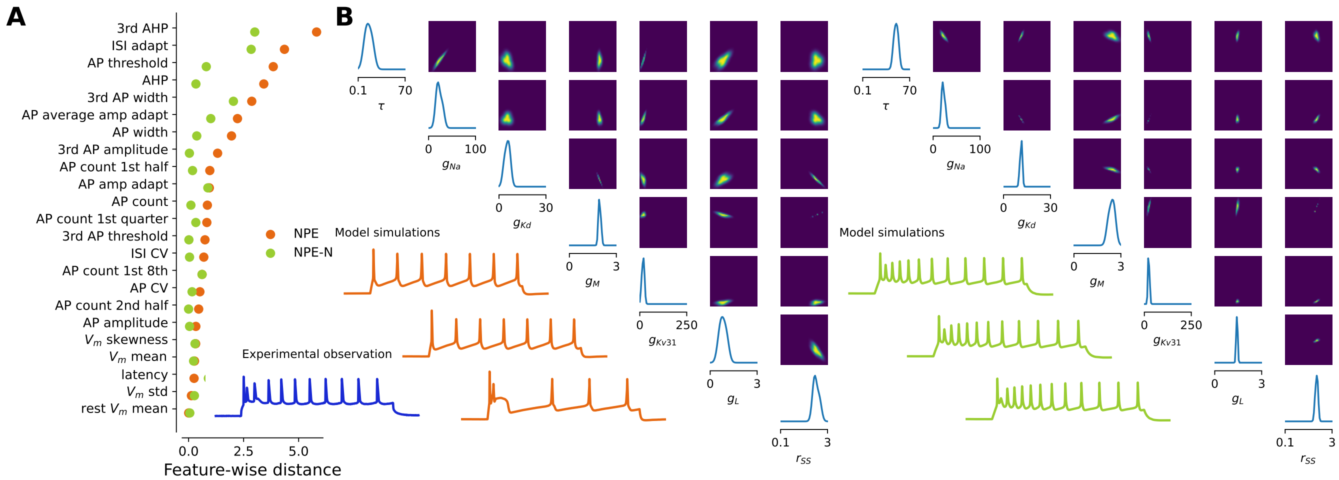

Figure S9 **NPE vs NPE-N, illustration 5: *L6 CT Cpa6* pyramidal cell.** Analogous to Fig. 3.

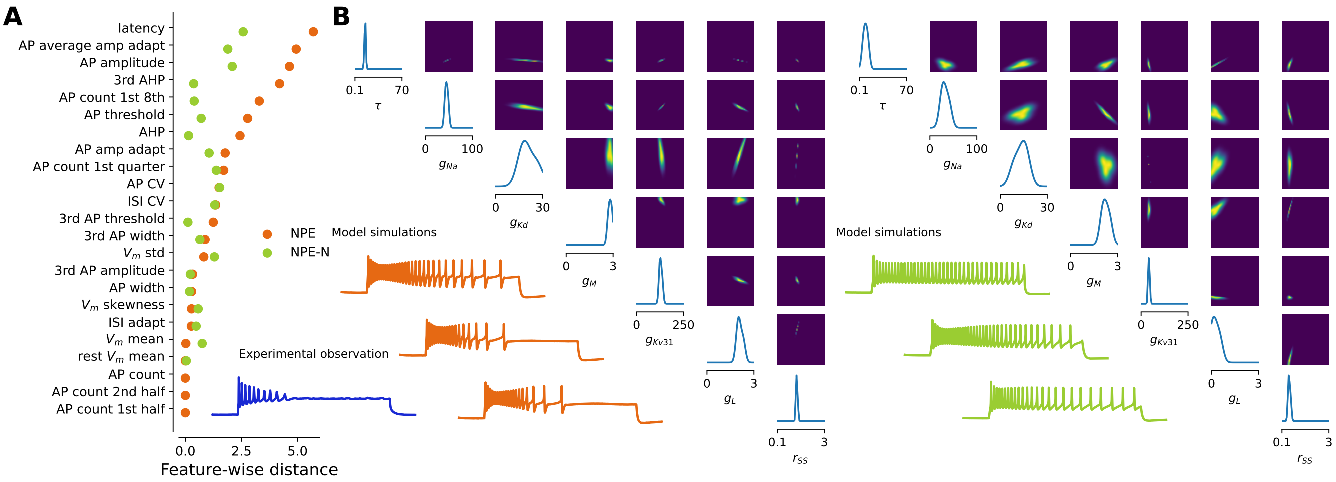

Figure S10 NPE vs NPE-N, illustration 6: *Sst Th_1* interneuron. Analogous to Fig. 3.

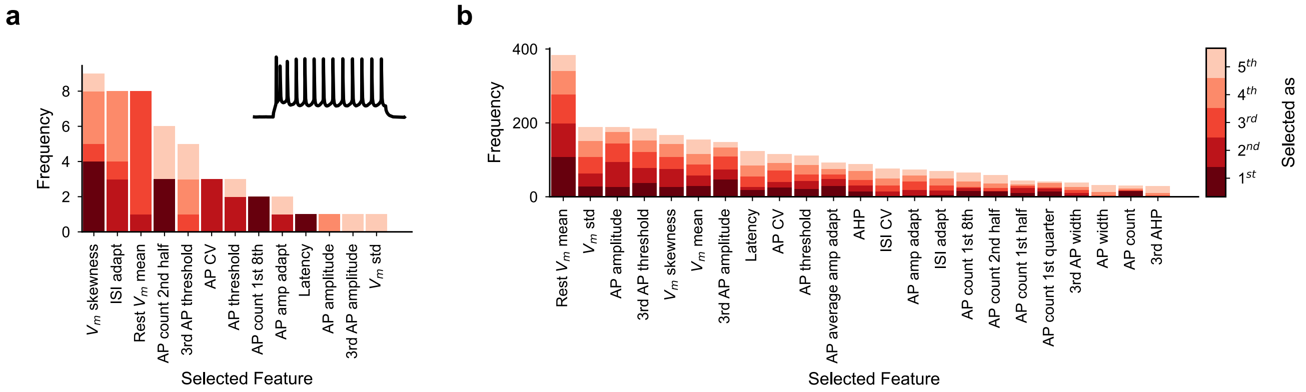

Figure S11 Ranking commonly used electrophysiological features by their ability to constrain posterior estimates. **a** Features are ranked by how often they are strongly constraining the posterior of a Pvalb neuron. Strongly constraining features minimize the KL divergence between posterior estimates subject to all 23 features and estimates considering only five. Important features were selected across 10 repeated runs. Shading indicates the order in which they are selected as part of the top five. Features are ranked in descending order. **b** Summary across all 955 MOp neurons, of which features are strongly constraining the posterior estimates.

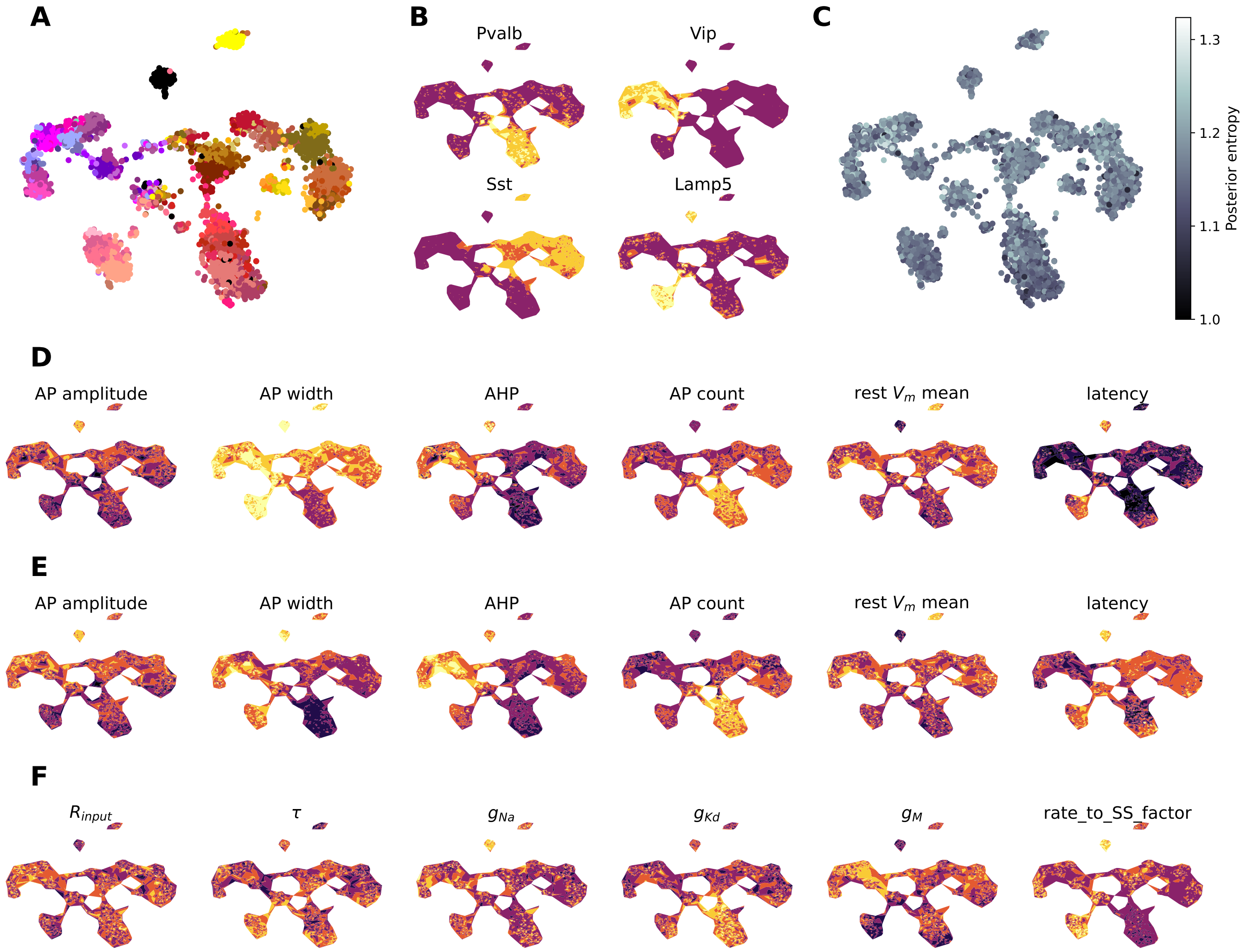

Figure S12 Two-dimensional embedding reveals difference in HH-based parameters between neural families. Analogous to Fig. 5, but for mouse visual cortex [16].

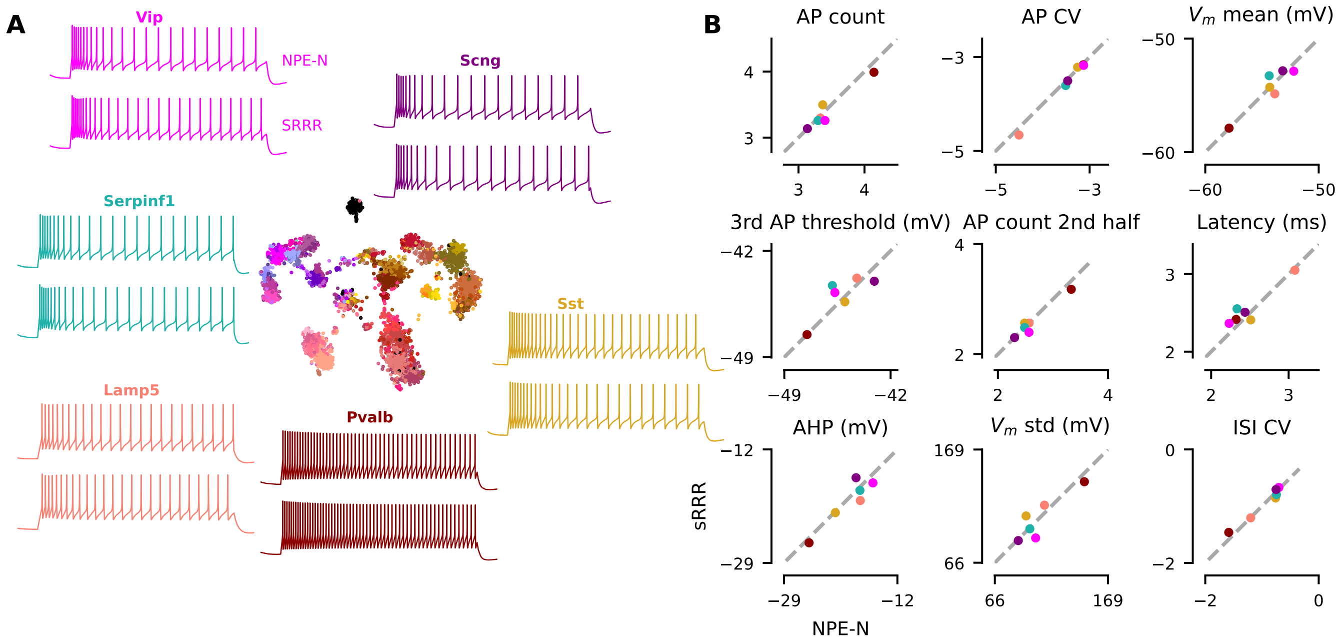

Figure S13 **Family representation of MAP estimates together with sRRR predictions** Analogous to Fig. 5, but for mouse visual cortex [16].

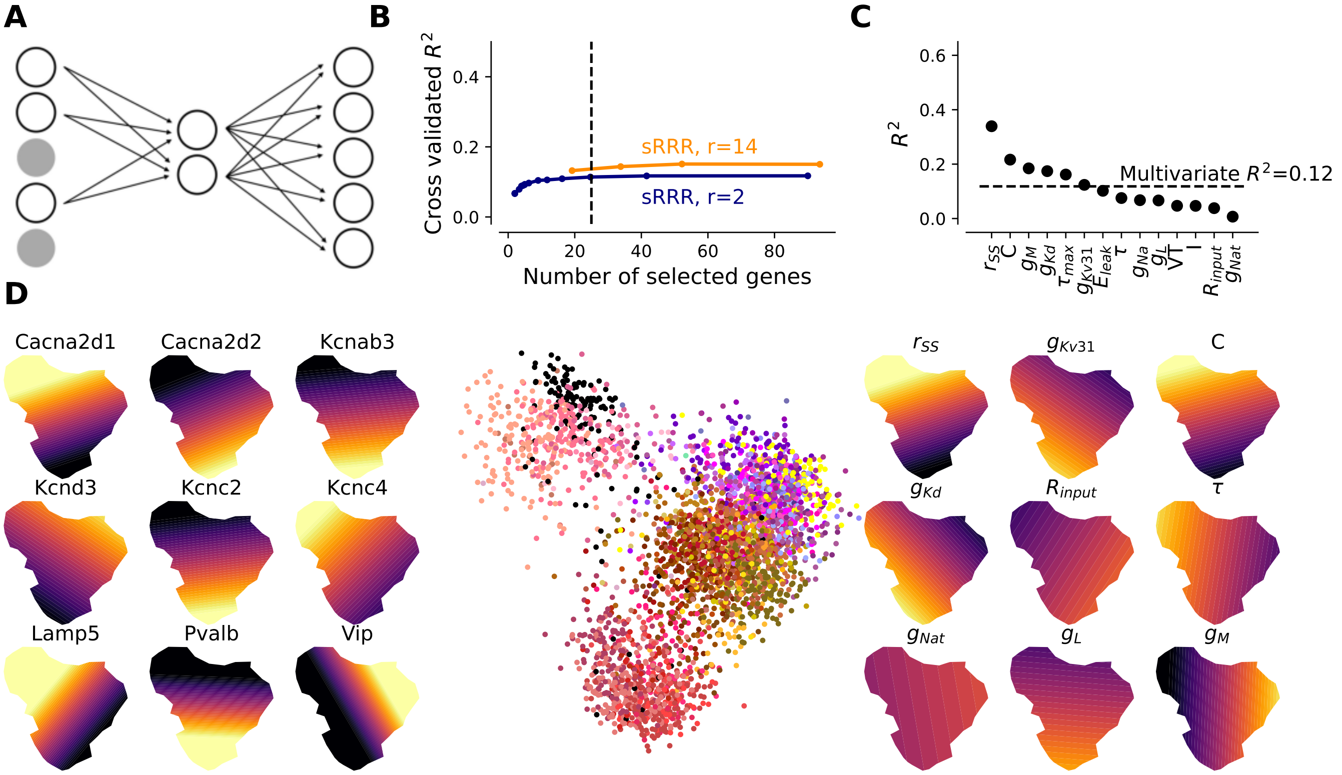

Figure S14 Prediction of MAP parameter estimates from gene expression with sparse reduced-rank regression. Analogous to Fig. 6, but for mouse visual cortex[16].

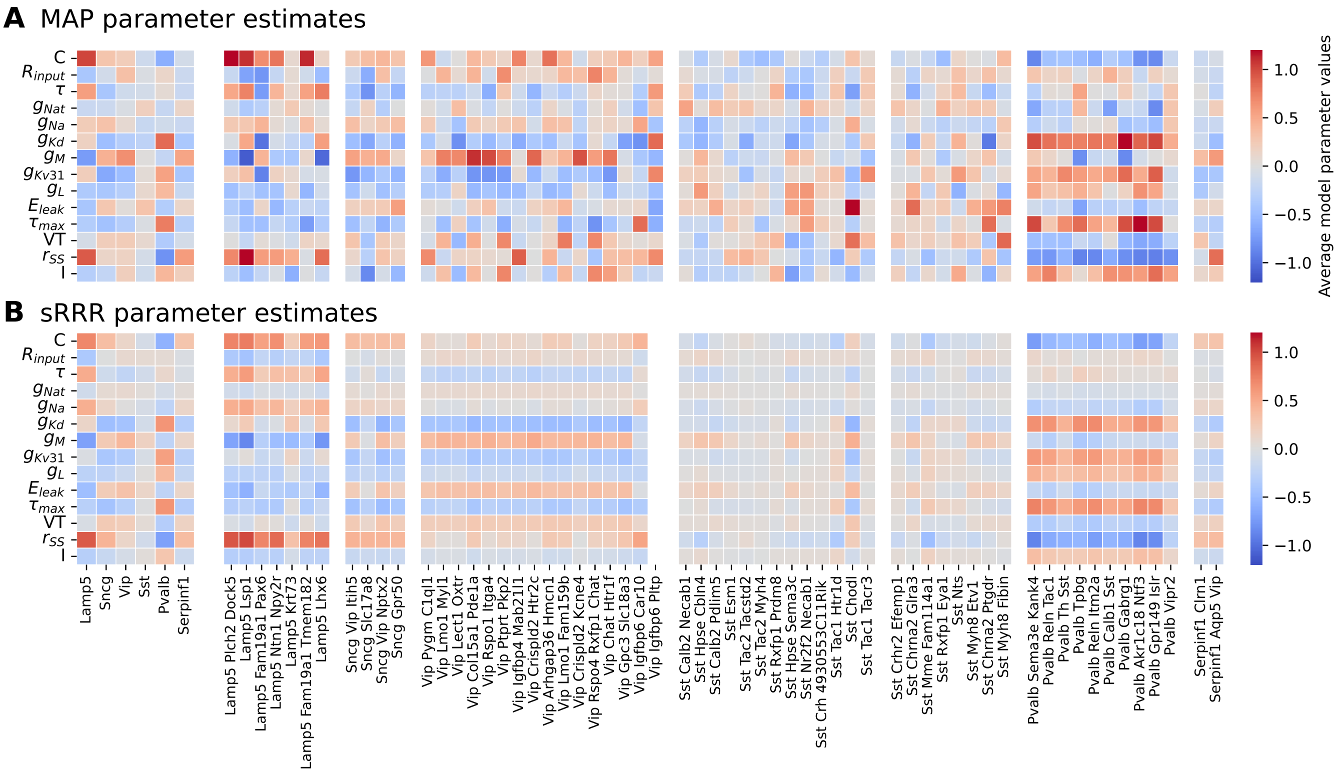

Figure S15 **MAP parameter estimates and sRRR predictions for each family and cell type.** Analogous to Fig. 7, but for mouse visual cortex [16]. We did not have sufficient cells representing *Vip Igfbp6 Pltp*, i.e. matching transcriptome and electrophysiology, leaving that column in **b** white.
